# First Symposium of “Grupo Español de Investigación en Vesículas Extracelulares (GEIVEX)”, Segovia, 8–9 November 2012

**DOI:** 10.3402/jev.v2i0.20256

**Published:** 2013-01-30

**Authors:** Francesc E. Borràs, Juan Manuel Falcón-Pérez, Antonio Marcilla, María Mittelbrunn, Hernando A. del Portillo, Francisco Sánchez-Madrid, María Yáñez-Mó

**Affiliations:** 1 Grupo Español de Investigacion en Vesiculas Extracelulares (GEIVEX); 2 IVECAT, IGTP, Badalona (Barcelona). Email: feborras@igtp.cat; 3 Metabolomics Unit. CIC bioGUNE, IKERBASQUE Foundation, Derio (Bizkaia). Email: jfalcon@cicbiogune.es; 4 Dpto. Biología Celular y Parasitologia, F. Farmacia, Universitat de Valencia, Valencia. Email: antonio.marcilla@uv.es; 5 Departamento de Biología Vascular e Inflamación, Centro Nacional de Investigaciones Cardiovasculares (CNIC), Madrid. Email: mmittelbrunn@cnic.es; 6 ICREA Barcelona Centre for International Health Research, (CRESIB, Hospital Clinic - Universitat de Barcelona). Email: hernandoa.delportillo@cresib.cat; 7 Departamento de Biología Vascular e Inflamación, Centro Nacional de Investigaciones Cardiovasculares (CNIC) and Servicio de Inmunología, Hospital de la Princesa, Instituto de Investigacion Sanitaria Princesa (IIS-IP), Madrid. Email: fsanchez.hlpr@salud.madrid.org; 8 Unidad de Investigación, Hospital Santa Cristina, Instituto de Investigación Sanitaria Princesa (IIS-IP), Madrid. Email: myanez.hlpr@salud.madrid.org

On the 8th and 9th of November, 71 scientists working in the field of extracellular vesicles from different laboratories in Spain met together in Segovia to present and discuss their research advances in this transformative and catalytic new field in translational biomedicine.

The symposium was started with opening and welcoming words from María Yáñez-Mó of Hospital Santa Cristina, Instituto de Investigación Sanitaria Princesa, Madrid, the main “driving force” behind this event. Afterwards, Hernando A. del Portillo, ICREA Research Professor at the Barcelona Centre for International Health Research – CRESIB, presented brief remarks on extracellular vesicles, the International Society of Extracellular Vesicles (ISEV) and the history of the “Grupo Español de Investigación en Vesículas Extracelulares” (GEIVEX). Particular attention was called upon the fact that some years ago, research in this area was largely neglected. Yet, with the discovery that extracellular vesicles can present antigens, transport miRNA and mRNA and act as inter-cellular communicators and as a result of their innovative and potential use in diagnostics and cancer therapies, research in this field has recently exploded. In fact, the number of publications on extracellular vesicles, as shown by the PubMed statistics under the query “extracellular vesicles”, has dramatically increased in recent years.

Research in this field received a major input with the creation of the International Society for Extracellular Vesicles (ISEV) whose consolidation and constitution was amended at the First Annual Congress of ISEV, held in Gothenburg, Sweden, in April of 2012. It was during this international Congress that the participating Spanish groups decided to launch a kick-off meeting in Barcelona to discuss the different possibilities for creating a task force on Extracellular Vesicles. This meeting was followed by two other meetings in Bilbao and Madrid, whose key actions were the constitution of the GEIVEX, the proposal of launching a theoretical/practical course on extracellular vesicles, and to host the first inclusive scientific symposium with other Spanish groups working in this field.

The symposium was hosted in Segovia (Spain), a city whose architecture, history and aqueduct (declared World Heritage by UNESCO) offered a unique environment for fostering discussions and synergisms. Organized as plenary lectures in six different sessions and one sponsor's technical session, the meeting hosted 71 participants from 21 laboratories from Spain, from different scientific backgrounds and interests (Figure 1). Below is a summary of the talks presented and whose outstanding scientific level, as illustrated in this summary, exceeded all expectations guaranteeing the future expansion of GEIVEX and the consolidation of new projects and collaborations among Spanish groups working in this new translational field.

**Fig. 1:**
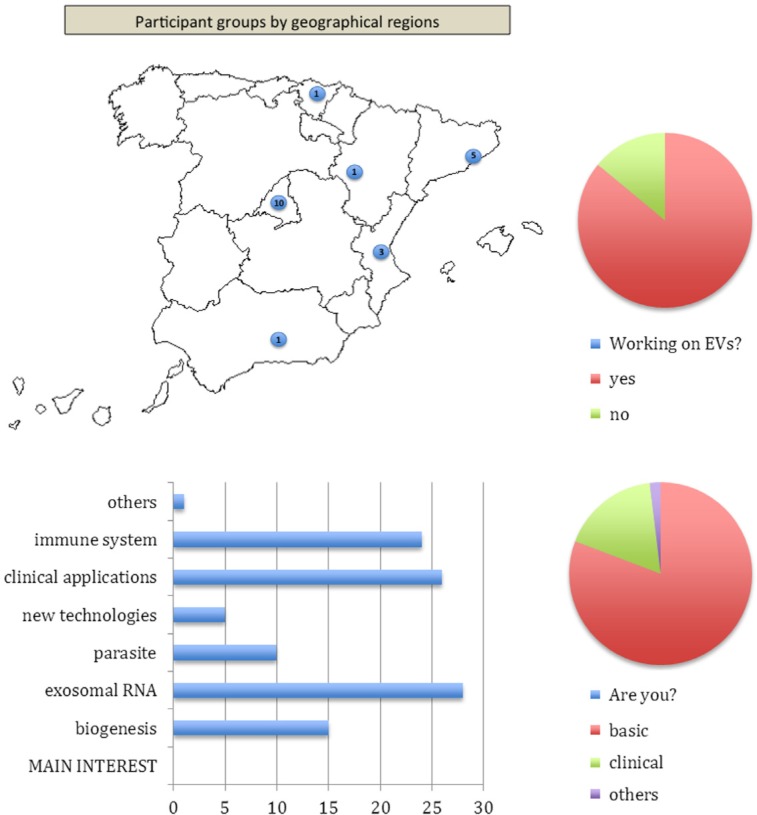
Profile of the participants in the GEIVEX symposium. Figure plots the geographical location of the participating groups, as well as their research background, interests and previous experience in EVs work. Bar chart depicts the number of participants expressing a particular interest.

## Session I: biogenesis of exosomes and sorting mechanisms

In the first session, chaired by Juan Manuel Falcón (CIC bioGUNE, Derio), three novel studies on the session “Biogenesis of exosomes and sorting mechanisms” were presented. These studies support the role of diacylglycerol kinase (DGK), MAL and some members of the tetraspanin family in the control of extracellular vesicles biology.

The group of Dr. Manuel Izquierdo (IIB, CSIC-UAM, Madrid) showed that after T lymphocyte activation the number of multivesicular bodies (MVBs) containing a Fas ligand increases significantly compared to naive lymphocytes. Importantly, the recruitment of DGKalpha to these MVBs exerts a negative role in the maturation process of MVBs affecting the secretion of exosomes. In addition, the interference in DGKalpha expression inhibits the polarization of MVBs towards the immune synapse and exosome secretion. Their results point to DGKalpha as an integration molecule for both positive and negative signals involved in exosome secretion during the immune adaptive response, and depending on their balance deciding the final cell response.

Leandro Ventimiglia, from the group of Miguel A. Alonso (Centro de Biología Molecular Severo Ochoa, CSIC-UAM, Madrid), presented data supporting the role of the protein MAL in the secretion of extracellular vesicles. MAL is a 17-kDa transmembrane protein identified as an essential component of the machinery for specialized exocytosis to the apical surface of epithelial MDCK cells and for transport of the tyrosine kinase Lck to the plasma membrane in T cells. Dr. Ventimiglia showed that MAL knockdown greatly impairs the secretion of CD59-positive exosomes in jurkat T cells, suggesting that MAL might play a role in the route of exosome generation.

Finally, María Yáñez-Mó (Hospital Santa Cristina, IIS-IP, Madrid) delivered a very interesting presentation on how the alterations in the expression of some members of the tetraspanin family – hallmarks of many extracellular vesicles – are reflected in the composition of tetraspanin-enriched membranes (TEM) and extracellular vesicles. For that purpose, they characterized the intracellular TEM interactome by high-throughput mass spectrometry, in both human lymphoblasts and their derived exosomes that revealed a clear pattern of interaction networks. Data obtained from quantitative proteomics showed that TEM ligands account for a great proportion of the exosome proteome demonstrating that insertion into TEMs is critical for correct protein sorting into the exosomes.

## Session II: RNA and extracellular vesicles

The second session, chaired by Francesc E. Borrás (IVECAT-IGTP, Badalona), included four presentations regarding the RNA content of microvesicles, their biological significance in the immune system and in reproductive biology, and some technical issues about their purification.

The group of Francisco Sanchez-Madrid from CNIC and Hospital La Princesa in Madrid, presented results regarding two important issues about RNA and their content in microvesicles. First, Carolina Villarroya shed some light on the elegant mechanism on the specific miRNA sorting to exosomes. They show that nucleotide specific sequences present in mature miRNAs are responsible for their specific cargo into exosomes. In a second study from the same group, María Mittelbrunn showed their results on the immune-synapsis dependent transfer of miRNAs through exosomes. Using an in vitro model, the authors demonstrated the transfer of CD63-labelled microvesicles from T cells to antigen presenting cells, but not in a reverse way. The actual significance of this finding and the consequences to the APC are yet to be clarified.

Juan Manuel Moreno, from the Fundación IVI in Valencia, presented the third talk. This group is interested in studying the role of microvesicles including exosomes and their associated miRNAs in the decidualisation process that permits the differentiation of endometrial stromal fibroblasts into decidual cells. The authors analysed the miRNA profile and found a group of up-regulated and down-regulated miRNAs in decidualized hESCs compared to non-decidualized hESCs. These results produced a signature of hESC decidualization, which may be of relevance in studies of human gynaecological disorders including endometriosis, pre-eclampsia and others that may provoke infertility or repeated abortion episodes.

Finally, Mireia Olivan, from the Vall d'Hebron Research Institute (VHIR) in Barcelona, presented results of a study conducted to standardize RNA and miRNA purification from urinary exosomes. Their main target is the identification of biomarkers for the early diagnosis of human prostate cancer. Indeed many other groups among the audience – especially those working on urinary microvesicles – expressed their interest in the results. Using commercially available reagents, the authors showed a series of experiments that concluded the usefulness of specific RNA kits, and confirmed the relative stability of nucleic acids in exosomes, including those obtained from freeze–thawed urine samples, thus permitting their use for biomarker discovery.

## Session III: parasite and viral extracellular vesicles

In this session, chaired by Maria Mittelbrunn (Centro Nacional de Investigaciones Cardiovasculares, Madrid), the presence and significance of exosomes in infectious parasitic and viral diseases were described.

Lorena Martin-Jaular, working at the Centre for International Health Research (Barcelona), in the group of Hernando A. del Portillo, presented data demonstrating that reticulocyte-derived exosomes from mice infected with malaria are involved in antigen presentation and in the induction of immune responses against the parasite. The presence of parasite proteins in these extracellular vesicles has been detected by proteomic analysis. Interestingly, Lorena showed that immunization of mice with reticulocyte-derived exosomes from malaria infection induces parasite-specific protective immune responses, which caused complete and long-lasting protection. Thus, these results indicate that reticulocyte-derived exosomes can be explored as a novel vaccine and platform against malaria.

Dolores Bernal, working with Antonio Marcilla in the Universitat de València (Valencia), demonstrated the secretion of exosome-like vesicles by parasitic helminths, specifically the trematodes *Echinostoma caproni* and *Fasciola hepatica*. These microvesicles are actively released by the parasites and can be taken up by host cells. Trematode extracellular vesicles contain most of the proteins previously identified as components of exosomes. In addition to parasitic proteins, host proteins were also detected in these vesicles. These data suggest an important role for exosomes in host–parasite communication.

Antonio Osuna, from Universidad de Granada (Granada), showed data on the detection and characterisation of extracellular vesicles from *Trypanosoma cruzi*. Their data indicate that the different forms of the parasite produce exosome-like vesicles. Moreover, both the surface of the parasite as well as the cell that was being parasitized bore vesicles that in size and morphology resemble exosomes. Immunogold labelling and electron microscopy revealed the presence of some proteins from the parasite such as DGF-1, telomerase, MASP proteins, prohibitine 1 and 2, actin, HSP70 and a “small like calpaine”. The detection of these extracellular vesicles from *T. cruzi* could be used as a diagnostic marker from Chagas patients.

Frederik Verweij, from VUmc Cancer Center Amsterdam, the Netherlands, demonstrated how the level of NF-κB activation by the oncoprotein latent membrane protein 1 (LMP1) encoded by the Epstein Barr virus was regulated by its trafficking through the endo-exosomal pathway. LMP1 is a viral mimic of CD40, but lacks classical regulatory features of the TNF receptor family as ligand control or intracellular degradation. This renders LMP1 a constitutive active protein and poses a risk of malignant conversion of the infected cell, as demonstrated by its association with different lymphomas and carcinomas. LMP1 associates with tetraspanin CD63 in a subset of MVEs. Impairment of exosomal secretion of LMP1 by GFP-fusion or CD63 knockdown drastically increased its NF-kB activation levels. This poses the trafficking and secretion of LMP1 via the endo-exosomal pathway as an alternative mechanism to antagonize constitutive NF-κB (over)activation.

## Session IV: clinical applications of extracellular vesicles

In the fourth session, chaired by Antonio Marcilla (Universitat de València), three studies on urine exosomes along with oral and hepatic exosomes were presented. In addition, an overview of current studies on polymer therapeutics was also presented.

Juan Manuel Falcón (CIC bioGUNE, Derio), whose group has been studying sub-cellular compartments as a source for extracellular vesicles for more than seven years, showed their application in the discovery of non-invasive tissue-specific disease biomarkers, focusing on liver diseases. They presented a thorough qualitative and quantitative analysis of the proteome of hepatocyte-derived extracellular vesicles challenged to different model toxins. By applying label free quantitation proteomics, they showed significant deregulation of a number of proteins from those vesicles after treatments, identifying candidate markers for liver injury. They also presented in vivo validation for some of those markers, supporting the suitability of that strategy to unravel non-invasive candidate biomarkers for disease. In addition, they presented proteomic characterization of extracellular vesicles purified from the plasma of healthy individuals showing a high inter-individual variability that can have an important impact for transfusion-based therapies.

Marina Rigau, from the group of Jaume Reventós (Vall d'Hebron Institute of Oncology, Vall d'Hebron Universitary Hospital, Barcelona), presented data where exosomes could be used for early diagnosis of prostate cancer. They showed that dithiothreitol (DTT) treatment followed by ultracentrifugation was the best method for isolating urinary exosomes. Preliminary proteomic analysis revealed a list of almost 100 proteins in those exosomes, including known markers for prostate cancer, demonstrating the usefulness of body fluids for extensive comparative proteomic studies and thus allowing for the discovery of new potential candidate markers for improving prostate cancer diagnosis.

Gloria Alvarez-Llamas (IIS-Fundación Jiménez Díaz, Madrid) showed the usefulness of exosomes in the diagnosis and follow-up of diabetic nephropathy (DN). She showed an optimum protocol for exosome isolation from human urine samples, including DTT treatment to enrich the exosomal fraction and, in particular, designing a strategy to deplete albumin – a high abundance protein in nephropathy patients. Differential proteomic analysis by label-free LC-MS/MS revealed 562 proteins identified in human urinary exosomes – 240 proteins previously unreported in those vesicles. A panel of three proteins was discovered to significantly respond to physiopathological changes in DN.

Ana Armiñan, from the group of Maria Jesus Vicent (Polymer Therapeutics Lab., Centro de Investigación “Príncipe Felipe”, Valencia), presented an interesting overview of current studies on polymer therapeutics and their usefulness as specific target treatments for different diseases like cancer and infectious diseases, or regenerative medicine, as well as for their use in the diagnosis of diseases as imaging tracers.

Patricia Carrasco, from the group of Miguel Quintanilla (Instituto de Investigaciones Biomédicas “Alberto Sols”, Madrid), showed the presence of podoplanin in exosomes. To investigate the role of podoplanin-containing exosomes in the tumorigenic potential of carcinoma cells, they carried out studies in a cell line derived from a human oral carcinoma, which expresses relatively high levels of podoplanin. Depletion of podoplanin protein levels by shRNA interference drastically reduces the tumourigenicity of HN5 cells in athymic nude mice.

Finally, Inés Lozano, from the group of Francesc E. Borrás (IVECAT-IGTP, Badalona, Barcelona) showed an interesting study where different protocols for exosome purification from urine samples were checked. These included different ultracentrifugation conditions as well as the use of different available commercial kits such as Vivaspin filtration. The methods used took into account the protein concentration, the concentration and size of the obtained vesicles and a brief phenotype. They showed that all three methods studied are suitable for the enrichment of microvesicles, although the composition of the sample obtained in each method reflects significant changes.

## Session V: extracellular vesicles in the immune system

The pivotal role played by extracellular vesicles in immune responses was presented in this session chaired by Francisco Sanchez-Madrid. Exosomes present antigen and regulate the activation and function of NK cells and macrophages. In addition, they may be suitable biomarkers for atherosclerosis of therapeutic agents for autoimmune diseases.

Francesc E. Borràs (IVECAT-IGTP, Badalona, Barcelona) presented data demonstrating the higher capacity of mature monocyte-derived dendritic cells (DCs) to internalize exosomes, despite being less phagocytic than immature DCs. As the capture of exosomes by DCs is of great relevance in transplantation, since exosomes bear MHCI and MHCII and can therefore present antigens, Dr. Borràs’ group studied the capacity of blood-borne DCs to capture exosomes. They showed that both conventional DCs and plasmacytoid DCs are capable of capturing exosomes, although the latter at a slower rate than conventional DCs. They also showed that pDCs can present exosomal antigens to autologous T cells. As this specialized DC subset has been previously implicated in the tolerogenic response to aloantigens, this study pointed to exosomes and other microvesicles as a source of antigen for DCs to induce tolerance.

Carlos Ernesto Fernandez, working in the IIS-FJD in the group of Jose Luis Martin-Ventura, presented novel data regarding the involvement of exosomes as a mechanism of secretion of novel biomarkers of atherosclerosis. Interestingly, the secretion of exosomes by vascular cells seems to be modulated by antioxidants, thus linking pathological pathways of atherosclerosis such as oxidative stress with the production of exosomes. Further studies would delineate the potential use of exosomes as biomarkers of atherosclerosis.

María del Mar Valés-Gómez (CNB, Madrid) centres her research on natural killer (NK) cells, which are a first line of defence to tumour and infection, whose cytotoxic activity is regulated by a balance of inhibitory and activating receptors. The activating receptor NKG2D is present in all human NK and T cells and mediates recognition of pathogen-infected and cancer cells. However, tumour cells can evade the immune system by releasing NKG2D ligands to induce down-regulation of the receptor. Some NKG2D-ligands can be recruited to exosomes and potently modulate receptor expression, while other are more susceptible to metalloprotease cleavage and shed as soluble molecules. The presence of NKG2D ligands in cancer patient sera can be used as a marker of tumour progression; for example, ULBP2 is found in advanced stages of melanoma. Nevertheless, the route of NKG2D-ligand release to the supernatant and their polymorphism are important factors to consider in the use of these proteins as cancer biomarkers.

Luis Martínez Lostao (Universidad de Zaragoza) showed the impressive potential of artificial liposomes tailored to bear TNF-related apoptosis inducing ligand (TRAIL) on their surface (LUVs-Apo2L/TRAIL). Apo2L/TRAIL is a death ligand found on exosomes secreted by activated T-lymphocytes. The authors constructed liposomes bearing a Ni^2+^quellating molecule to bind in a correct conformation the His-tagged TRAIL recombinant protein to resemble natural exosomes. LUVs-Apo2L/TRAIL was shown to be a more effective treatment in rheumatoid arthritis than soluble Apo2L/TRAIL without showing hepatotoxic effects.

Agnieszka Koziol (CNIC, Madrid) showed that the macrophage secretome contained 425 proteins, 71% of which were of exosomal origin. Among them, lactadherin (milk fat globule-EGF factor 8, or MFGE8) appeared to be a putative substrate of MT1-MMP proteolytic activity, which was confirmed by in vitro digestion assay. A MT1-MMP-dependent cleavage site is located at the phosphatidilserine-binding motif responsible for binding to exosomes and apoptotic cells so that MT1-MMP may modify the binding properties of MFGE8. MFGE8 KO presents a defect in the clearance of apoptotic cells and immune resolution leading to the development of autoimmune diseases. To determine whether MT1-MMP is involved in this function of MFGE8, they analysed the efficiency of MT1 −/− and MT1 +/− mice in apoptotic cell clearance. Both MT1−/− and MT1 + /− displayed defects in phagocytosis of apoptotic cells. MT1 + /− mice also showed reduced survival in an experimental sepsis model and developed an autoimmune disease because of impaired immune response resolution.

## Sponsoring session: New technologies in extracellular vesicle research

Our sponsors presented their own claimed solutions for extracellular vesicle research in this appealing session chaired by Maria Yañez-Mo.

Ben Owen, from NanoSight, introduced their already widely used equipment, based on nanoparticle tracking, which, based on their Brownian coefficient, determines the size of particles in solution and their concentration.

This technology also allows for the fluorescent labelling of the vesicles. Miltenyi specialist Dr. Alexander Adan commented on the special characteristics of their cytometer, which make it particularly well suited for the study of extracellular vesicles. Other than its small size, it has a volumetric counter and can determine the relative size of nanoparticles in the side scatter, which has a broader angle (15°) than other cytometers. Moreover, they are currently working on adapting their famous MACs system to the isolation and purification of extracellular vesicles.

Dr. Dimitri Aubert, from Izon Science, presented the qNano system for high accuracy extracellular vesicle characterisation and quantification, based on their patented tunable resistive pulse sensing technology. This non-optical method counts every particle going through a size-tunable nanopore and measures the resulting changes in conductivity, giving very accurate measurements for particles of diameters 40 nm to 10 µm.

Finally, Isabel Crespo, responsible of the cytometry platform of IDIBAPS and invited by BD, presented flow cytometry data on microvesicles. She prepared a very comprehensive report on the requirements for accurate detection of these very small particles, such as buffer filtration, low flow rate, and thresholding to discriminate noise (based on fluorescence by annexin labelling in this case, or even FCS/SSC). Flow cytometry of extracellular vesicles is a future challenge to discriminate by specific markers among the heterogeneity of extracellular vesicles in our preparations from biological samples, which is pushed to the limit by the small size and scarcity of some antigens in extracellular vesicles.

## Concluding remarks

The first scientific symposium of GEIVEX bringing together Spanish groups working on different aspects of extracellular vesicles surpassed all expectations as judged by the evaluation made by the participants (see Figure 1). Overall, the meeting, the venue, the organisation, the format, the quality and the topics were all highly appreciated. In addition, several collaborations and future plans were, respectively, created and discussed. Among them, it is worth mentioning the next two key actions of GEIVEX: (1) an international workshop on extracellular vesicles to be held at the “Menéndez Pelayo” International University on 18–20 September 2013 (see http://geivex.wordpress.com/3-courses/uimp-course-vesiculas-extracelulares-implicaciones-en-biomedicina/); and (2) the next meeting which will be hosted in in Catalunya in the first or second trimester of 2014. Finally, the organizers would like to thank all of the speakers, participants and sponsors for making this first meeting of GEIVEX possible.

